# Prognostic and immunological role of Ras-related protein Rap1b in pan-cancer

**DOI:** 10.1080/21655979.2021.1955559

**Published:** 2021-08-04

**Authors:** Guoliang Cui, Can Wang, Zhenyan Lin, Xiaoke Feng, Muxin Wei, Zhengyue Miao, Zhiguang Sun, Fei Wei

**Affiliations:** a Department of Gastroenterology, The Second Affiliated Hospital of Nanjing University of Chinese Medicine, Nanjing, China; b Department of Traditional Chinese Medicine, The First Affiliated Hospital of Nanjing Medical University, Nanjing, China; c Institute of Integrated Chinese and Western Medicine, Nanjing Medical University, Nanjing, China; d Department of Physiology, School of Medicine & Holistic Integrative Medicine, Nanjing University of Chinese Medicine, Nanjing, China

**Keywords:** Rap1b, pan-cancer, survival analysis, prognostic biomarker, immune inﬁltration, tumor microenvironment

## Abstract

Ras-related Protein *Rap1b*, a GTP-binding protein belonging to the proximal RAS, which affects tumor progression through regulating tumor cell proliferation, invasion and participates in the functions of various immune cells. However, the potential roles and mechanisms of *Rap1b* in tumor progression and immunology remains unclear. In this study, we systematically analyzed the pan-cancer expression and prognostic correlation of *Rap1b* based on GTEX, CCLE, Oncomine, PrognoScan, Kaplan–Meier plotters and TCGA databases. The potential correlations of Rap1b with immune infiltration were revealed via TIMER and TCGA database. SangerBox database was used to analyzed the correlations between *Rap1b* expression and immune checkpoint (ICP), tumor mutational burden (TMB), microsatellite instability (MSI), mismatch repairs (MMRs) and DNA methylation. The results indicated that the expression level of *Rap1b* varies in different tumors. Meanwhile, the expression level of *Rap1b* strongly correlated with prognosis in patients with tumors, higher expression of *Rap1b* usually was linked to poor prognosis in different datasets. *Rap1b* was correlated closely with tumor immunity and interacted with various immune cells in different types of cancers. In addition, there were significant positive correlations between Rap1b expression and ICP, TMB, MSI, MMRs and DNA methylation. In conclusion, the results of pan-cancer analysis showed that the abnormal *Rap1b* expression was related to poor prognosis and tumor immune infiltration in different cancers. Furthermore, *Rap1b* gene may be used as a potential biomarker of clinical tumor prognosis.

## 1 Introduction

According to global cancer statistics, there will be about 1.2 million new cancer cases and 400,000 cancer deaths worldwide in 2020 compared with 2018 [1,2]. There occurs a profound impact on countries around the world with the rapidly growing burden of cancer [3]. Therefore, effective prevention and monitoring measures for the incidence and development of tumors, searching for the key targets of cancer therapy have become hot issues in current research.

Ras-related Protein *Rap1b*, a GTP-binding protein associated with the Ras family [4], is widely expressed in various human tissues and affects a variety of cell functions, containing regulation of cell proliferation and differentiation, migration and polarity [5], enhancement of endothelial cell adhesion [6], maintenance of vascular endothelial permeability [7]. In addition, Rap1b had been reported to be involved in the development and progression of a variety of malignant tumors. By inhibiting the expression of Rap1b, the proliferation and migration of hepatocellular carcinoma (HCC) [8], renal cell carcinoma (RCC) [9], esophageal squamous cell carcinoma (ESCC) [10], colorectal cancer cells (CRC) [11] and melanoma cells [12] had been inhibited. Moreover, downregulation of *Rap1b* inhibited the invasion of thyroid cancer (TC) and epithelial mesenchymal transformation (EMT) [13,14], whereas the autophagy and apoptosis in gastric cancer cell lines MKN-28 and SGC-7901 had been promoted [15]. These studies revealed that the expression level of *Rap1b* was related to tumor proliferation, invasion, and migration. However, human pan-cancer evidences regarding the potential function of Rap1b in various tumors remains unclear.

The interaction between immune cells and tumor cells in the tumor microenvironment performed a crucial role in tumor growth, invasion and metastasis [16,17]. *Rap1b* had a regulatory effect on immune cells, regulating the development and maturation of B lymphocytes and T cell-dependent humoral immunity [18]. *Rap1b*was involved in neutrophils migration, and downregulation of Rap1b promoted migration and recruitment of inflammatory pulmonary neutrophils [19]. Besides, *Rap1b* maintained normal lymphatic development, barrier function and tissue permeability [20]. Therefore, we speculate the effect of *Rap1b* on the progression of various tumors was related to tumor immune infiltration. In addition, the roles of Rap1b in immunology in pan-cancer has seldomly been analyzed systematically.

In this study, we systematically analyzed the expression level of *Rap1b* and its prognostic correlations, using Genotype-Tissue Expression (GTEx), Cancer Cell Line Encyclopedia (CCLE), Oncomine, PrognoScan, Kaplan–Meier plotters and The Cancer Genome Atlas (TCGA) databases. We then investigated the potential correlations of Rap1b expression with immune infiltration based on the Tumor Immune Estimation Resource (TIMER). Moreover, we comprehensively analyzed the association between *Rap1b* expression and ICP, TMB, MSI, MMRs and DNA methylation. Our findings indicated statistical correlations of *Rap1b* expression with clinical prognosis immune infiltration and genetic mutation, which suggests that Rap1b is a potential prognostic biomarker.

## 2 Materials and Methods

### Gene expression analysis of Rap1b

To compare the expression level of *Rap1b* gene in normal and tumor tissues, Rap1b gene expression in 31 normal human tissues and 21 tumor cells were obtained from Genotype-Tissue Expression (GTEx) portal (https://gtexport.org/home/) and Cancer Cell Line Encyclopedia (CCLE) database (https://portals.broadinstitute.org/ccle/about) [21]. The expression of *Rap1b* was also analyzed in 33 tumor and adjacent tissues in The Cancer Genome Atlas (TCGA) database (https://cancergenome.nih.gov/) [22] and the abbreviations and full names of 33 tumors were shown in Table 1. R software package (R version: 3.6.2) was applied to integrate the above database data, and the value of log2 (TPM+1) was converted to represent the gene expression level of Rap1b. The Oncomine database (https://www.oncomine.org/resource/login.html), TIMER database (https://cistrome.shinyapps.io/timer/) were used to compare the expression of Rap1b between tumor tissues and adjacent tissues [23]. In the Oncomine database, we set the threshold of P-value 0.05 and fold change 1.5.

**Table 1.** Abbreviations and full names of 33 tumors in TCGA database

Abbreviation	Full name
ACC	Adrenocortical carcinoma
BLCA	Bladder urothelial carcinoma
BRCA	Breast invasive carcinoma
CESC	Cervical squamous cell carcinoma
CHOL	Cholangiocarcinoma
COAD	Colon adenocarcinoma
DLBC	Diffuse large B cell lymphoma
ESCA	Esophageal carcinoma
GBM	Glioblastoma multiforme
HNSC	Head and neck squamous cell carcinoma
KICH	Kidney chromophobe
KIRC	Kidney renal clear cell carcinoma
KIRP	Kidney renal papillary cell carcinoma
LAML	Acute myeloid leukemia
LGG	Brain lower grade glioma
LIHC	Liver hepatocellular carcinoma
LUAD	Lung adenocarcinoma
LUSC	Lung squamous cell carcinoma
MESO	Mesothelioma
OV	Ovarian serous cystadenocarcinoma
PAAD	Pancreatic adenocarcinoma
PCPG	Pheochromocytoma and paraganglioma
PRAD	Prostate adenocarcinoma
READ	Rectum adenocarcinoma
SARC	Sarcoma
SKCM	Skin cutaneous melanoma
STAD	Stomach adenocarcinoma
TGCT	Testicular germ cell tumors
THCA	Thyroid carcinoma
THYM	Thymoma
UCEC	Uterine corpus endometrial carcinoma
UCS	Uterine carcinosarcoma
UVM	Uveal melanoma

## Prognostic value analysis of Rap1b

Cox regression analysis was performed to test the correlations between *Rap1b* expression and patients’ overall survival (OS), disease-specific survival (DSS), and disease-free interval (DFI) in each cancer type via using TCGA [22]. Forest Plot was used to represent the hazard ratio and 95% confidence interval between *Rap1b* and patients’ OS, DSS and DFI. The high and low expression levels were divided by the median of the expression level of Rap1b, and the corresponding relationship curve was further plotted in the R environment. PrognoScan (http://dna00.bio.kyutech.ac.jp/PrognoScan-cgi/PrognoScan.cgi) microarray datasets were used to examine the relationships of *Rap1b* expression levels with prognosis [24]. The threshold was adjusted to Cox P-value <0.05. Kaplan–Meier Plotter is a relatively comprehensive online tool that can be used to analyze the effects of 54,675 genes on survival in 21 cancer types. We used the Kaplan–Meier plotter to obtain the relationship between the Rap1b gene and OS and relapse-free survival (RFS) in 21 cancers. Hazard ratios (HRs) with corresponding 95% confidence intervals (CIs) and log-rank P-values were calculated. The prognostic value was considered statistically significant when the P-value was less than 0.05.

## Correlation between Rap1b expression and immune infiltration

The TIMER database (http://timer.cistrome.org/) includes the infiltration of six types of immune cells (B cells, CD4 + T cells, CD8 + T cells, neutrophils, dendritic cells and dendritic cells) from 10,897 tumor samples currently available in the TCGA database. We searched and analyzed the correlation between Rap1b and immune infiltration by means of TIMER, and downloaded and saved the data.

**Correlation between Rap1b expression and Immune Checkpoint (ICP) Genes, Tumor Mutational Burden (TMB), Microsatellite Instability (MSI), mismatch repairs (MMRs) and DNA methylation.**

SangerBox website (http://sangerbox.com/Tool) [25], which is a useful online platform for TCGA data analysis, was used to explore relationship between *Rap1b* expression and ICP, TMB, MSI, MMRs and DNA methylation. We collected the expression levels of 47 ICP genes and revealed the correlation between *Rap1b* and ICP genes by Cox regression analysis. Spearman’s rank correlation coefficient was used to describe the relationship between the expression levels of Rap1b, TMB and MSI in different types of tumors. The association between *Rap1b* expression, MMRs genes (*MLH1, MSH2, MSH6, PMS2* and *EPCAM*) and DNA methyltransferase was evaluated using Pearson test. *P* value less than 0.05 was considered statistically significant.

## Results

### Rap1b expression in pan-cancer

**Figure 1.**
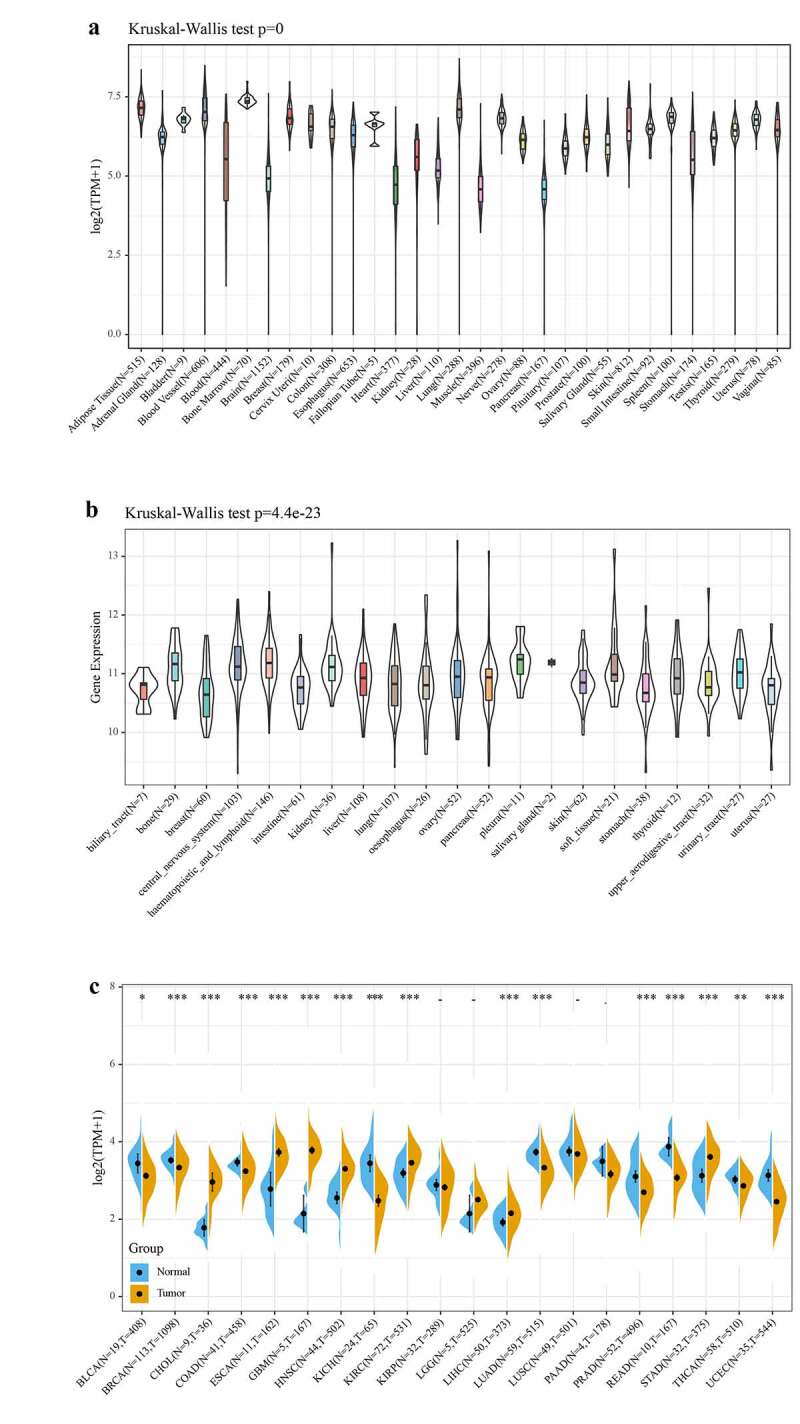
The expression level of Rap1b in different tumors. The expression of Rap1b in different normal tissues from GTEx database (a) and in different tumor cell lines from CCLE database (b). (c) The expression status of Rap1b gene in different cancers or specific cancer subtypes was determined by TCGA. *P < 0.05, **P < 0.01, ***P < 0.001

Firstly, we analyzed the data of 31 normal human tissues in GTEx database, and the results showed that the expression levels of *Rap1b* were similar in different normal human tissues, mainly concentrated in the value range of 3.75–7.5 (Figure 1a). The gene expression level of *Rap1b* in 21 tumor cell lines of CCLE database was generally higher than 10 (Figure 1b). The TCGA database data indicated that except for KIRP, LGG, LUSC, and PAAD, the expression of *Rap1b* in other 16 tumors and their corresponding adjacent normal tissues had statistically significant differences. The expression of *Rap1b* in 7 tumors (CHOL, ESCA, GBM, HNSC, KIRC, LIHC, and STAD) is higher than that in adjacent normal tissues. However, in 9 tumors (BLCA, BRCA, COAD, KICH, LUAD, PRAD, READ, THCA, and UCEC), the expression was lower than that in adjacent tissues (Figure 1c). Furthermore, the pan-cancer expression of *Rap1b* was examined by Oncomine and TIMER2 database. *Rap1b* expression was higher in cancer groups compared with the respective normal groups, including bladder, brain, cervical, esophageal, gastric, head, and neck, kidney, pancreatic and sarcoma cancer. However, the expression of *Rap1b* was found in breast, colorectal, leukemia, lung, ovarian and prostate was decreased. Moreover, we verified the expression results of *Rap1b* in TCGA by using TIMER2 database which based on the RNA sequencing data, and the results were consistent (Supplementary Figure 1).

## Multifaceted Prognostic Value of Rap1b

**Figure 2.**
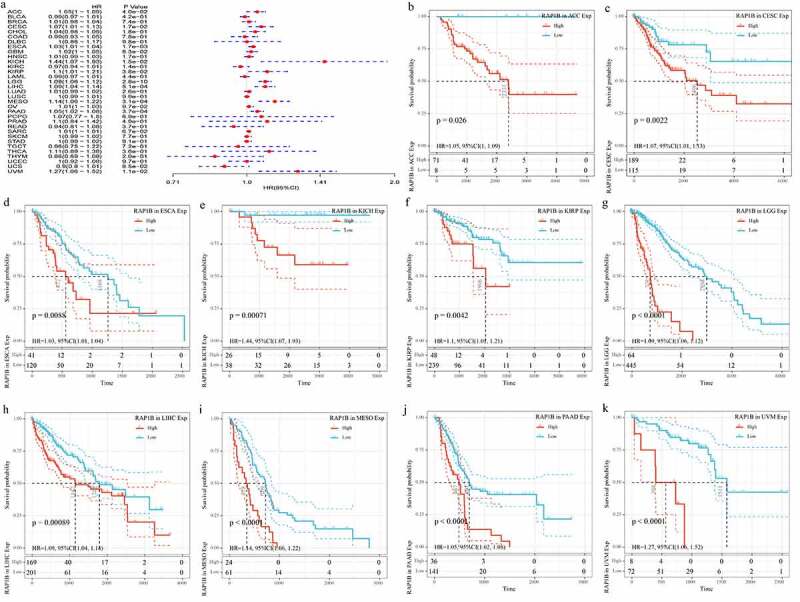
**Correlation between Rap1b expression with OS from TCGA database**. (a) Univariate Cox regression analysis and forest plot showed the hazard ratios related to *Rap1b* expression in pan-cancer. Red squares represent hazard ratio. Kaplan–Meier plotted survival curves of high and low expression of *Rap1b* in pan-cancer from TCGA database. OS of ACC (b), CESC (c), ESCA (d), KICH (e), KIRP (f), LGG (g), LIHC (h), MESO (i), PAAD (j), UVM (k). *P* < 0.05 is considered to be significant, with a 95% confidence interval of the dotted line. OS, overall survival

**Figure 3.**
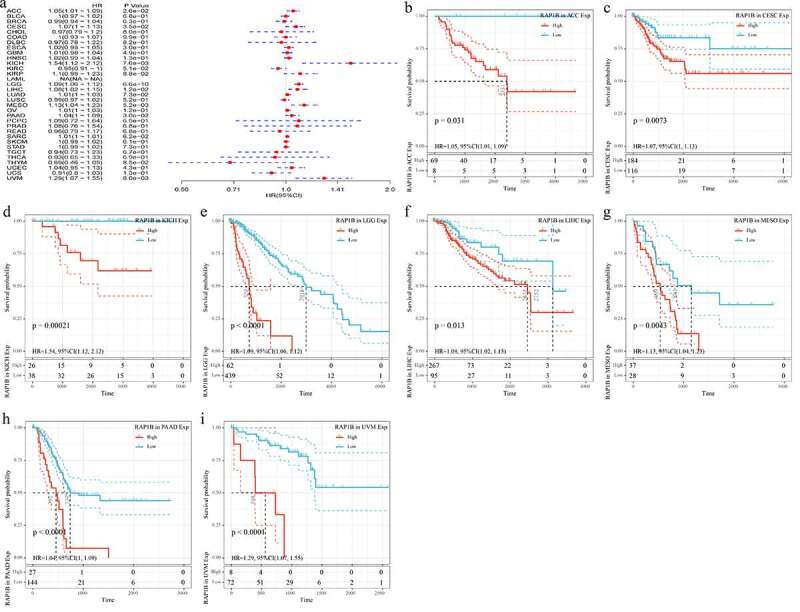
**Correlation of Rap1b expression with DSS from TCGA database**. (a) Univariate Cox regression analysis and forest plot showed the hazard ratios related to *Rap1b* expression in pan-cancer. Red squares represent hazard ratio. Kaplan–Meier survival curves comparing the high and low expression of *Rap1b* in different types of cancer from TCGA database. DSS of ACC (b), CESC (c), KICH (d), LGG (e), LIHC (f), MESO (g), PAAD (h), UVM (i). *P* < 0.05 is considered to be significant, with a 95% confidence interval of the dotted line. DSS, disease-specific survival

The results of GTEX, CCLE, and TCGA databases had showed that the expression level of *Rap1b* was different in tumors, but the prognostic value of *Rap1b* remained unclear. Therefore, we further analyzed whether the differential expression level of Rap1b was related to the prognosis of cancer patients. This study applied univariate Cox regression analysis and evaluated the correlation between Rap1b expression, OS, DSS and DFI in different tumors using TCGA database. As shown in Figure 2a, the risk ratios of Rap1b in ACC, CESC, ESCA, GBM, KICH, KIRP, LGG, LIHC, MESO, PAAD and UVM were significant, among which Rap1b had the highest risk effect on KICH (HR = 1.44, 95% CI from 1.07 to 1.93, *P* = 0.015), followed by UVM (HR = 1.27, 95% CI from 1.08 to 1.52, *P* = 0.011) and MESO(HR = 1.11, 95% CI from 1.07 to 1.93, *P* = 0.00031). Moreover, higher expression of *Rap1b* was positively correlated with the lower OS in ACC, CESC, ESCA, KICH, KIRP, LGG, LIHC, MESO, PAAD, UVM (Figure 2b-k). In the analysis of the correlation between DSS and Rap1b expression in different cancers, 8 cancer types showed the significant relationship. However, the risk ratio of Rap1b to LAML could not be calculated due to the lack of relevant data (Figure 3a). The analysis results of DSS and OS were similar, the higher expression of *Rap1b* positively correlated with lower DSS in ACC, CESC, KICH, LGG, LIHC, MESO, PAAD, and UVM (Figure 3b-i). Using PrognoScan and Kaplan–Meier Plotter, we analyzed the prognostic role of Rap1b in each cancer type and the results are shown in Supplementary Figure 2. Therefore, these results suggested that *Rap1b* expression is an independent risk factor for poor prognosis in these cancers.

## Immune cell infiltration of Rap1b in patients with cancer

**Figure 4.**
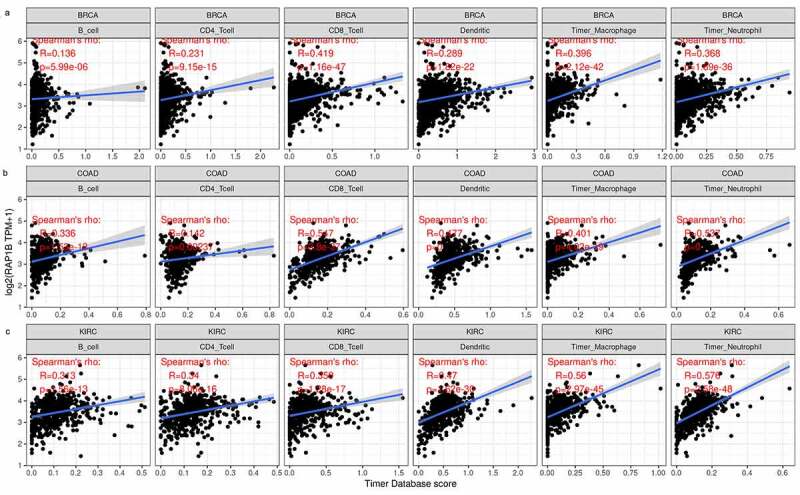
**Correlation of Rap1b expression with immune infiltration level in the top three tumors (BRCA, COAD, KIRC)**. Correlation between Rap1b expression and immune infiltration level in BRCA (a), COAD (b), KIRC (c)

Previous studies have shown that *Rap1b* has a regulatory effect on lymphocytes and neutrophils, therefore we searched the relationship between the expression of *Rap1b* and immune cell infiltration in tumors from TIMER database. According to the immune infiltration score criteria of 6 immune cell types (B cells, CD4 + T cells, CD8 + T cells, neutrophils, dendritic cells) in TIMER, we obtained a linear regression diagram through data analysis, and the results showed that the Rap1b expression in the majority of tumors was positively correlated with the level of infiltration of immune cells. In addition, *Rap1b* expression had significant correlation with infiltrating levels of B cells in 21 types of cancer, CD4 + T cells in 18 types of cancer, CD8 + T cells in 24 types of cancer, neutrophil in 27 types of cancer, macrophage in 26 types of cancer and dendritic in 26 types of cancer (Supplementary Figure 3). Overall, in three types of cancer (BRCA, COAD, and KIRC), *Rap1b* expression showed the most relevant to immune infiltration levels (Figure 4a-c). These findings strongly suggested that *Rap1b* affects patient survival through the interactions with immune cell infiltration in various cancers.

## Correlations of *Rap1b* expression with immune checkpoint genes

**Figure 5.**
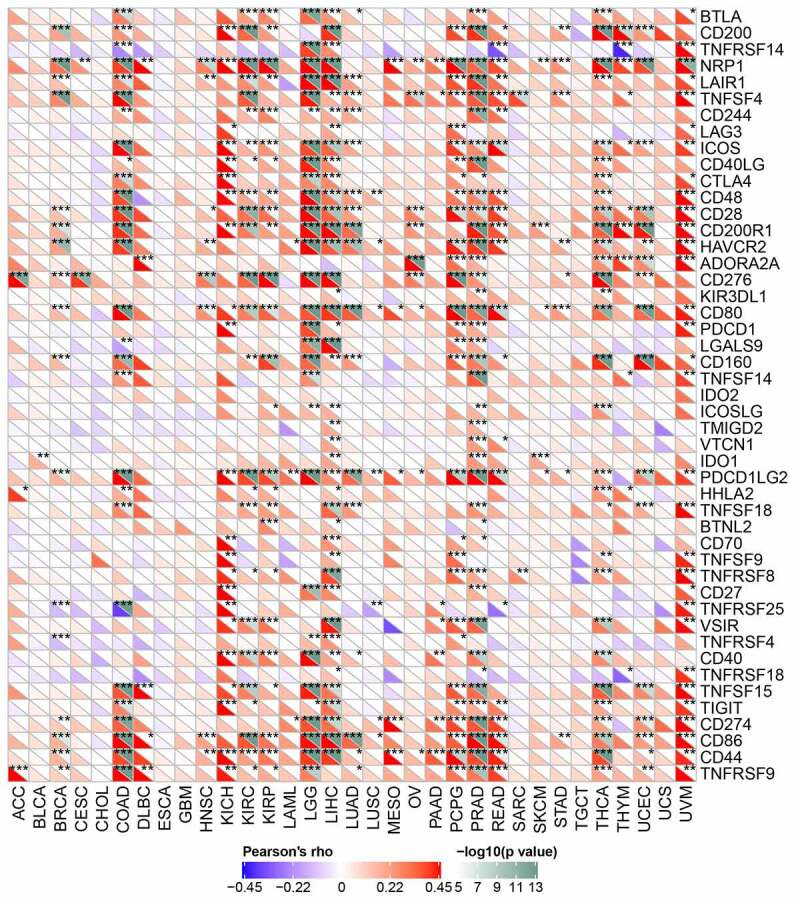
**Relationships between Rap1b expression and immune checkpoint gene**. The lower triangle of each tile represents the coefficient calculated by Pearson correlation test, and the upper triangle represents the *P-*value after log10 transformation. *P < 0.05; **P < 0.01; ***P < 0.001

The relationship between *Rap1b* expression and checkpoint gene expression in tumor immune response was further analyzed from mRNA sequence database. We found that *Rap1b* expression had a close link with T lymphocyte-related immune genes (CD80, CD86, CD28), neutrophil associated immune genes (CD44) and cancer-related genes, such as neuropilin-1 (NRP1), endothelial low-affinity A2b adenosine receptor (ADORA2A) and programmed cell death 1 ligand 2 (PDCD1LG2) in different kinds of tumors. Additionally, *Rap1b* was co-expressed significantly with more than 20 immune checkpoint genes in COAD, KICH, KIRC, KIRP, LGG, LIHC, PCPG, PRAD, THCA and UVM. These results suggesting that Rap1b expression was positively correlated with the expression of immune checkpoint genes in various tumors and *Rap1b* perhaps regulated tumor immune response by immune checkpoint regulation. However, *Rap1b* was negatively correlated with multiple immune checkpoints in CHOL and ESCA, but there was not significant of the negative correlation (Figure 5).

## Correlations of Rap1b expression with TMB and MSI

**Figure 6.**
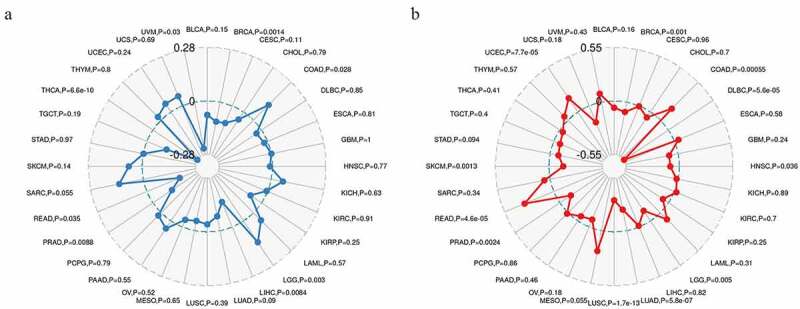
**Relationship between Rap1b expression, TMB and MSI in pan-cancer**. (a) The relationship between TMB and Rap1b. (b) The relationship between MSI and Rap1b. Spearman rank correlation test, *P* < 0.05 was considered statistically significance

TMB and MSI are important biomarkers of immunotherapy and have clinical practical value [20]. Hence, we calculated the TMB and MSI of each sample in all tumors and explored the relationship between *Rap1b* expression, TMB and MSI in different cancer types using Spearman rank correlation test. In the data of 32 cancers, the expression of Rap1b in 8 cancers was significantly correlated with TMB, including THCA, BRCA, LGG, READ, LIHC, PRAD, COAD, UVM (Figure 6a). The results also indicated that *Rap1b* expression was positively correlated with the high mutation status in LGG and COAD, and with the low mutation in THCA, BRCA, READ, LIHC, PRAD, and UVM. In addition, there are significant correlations between the expression of *Rap1b* with MSI in 11 cancer types, including LUSC, LUAD, READ, DLBC, UCEC, COAD, BRCA, SKCM, PRAD, HNSC, LGG (Figure 6b). The *Rap1b* expression was positively correlated with the MSI in READ, UCEC, and COAD, while negatively correlated with the MSI in LUSC, LUAD, DLBC, SKCM, PRAD, HNSC, and LGG. Based on the analysis results of TMB and MSI, Rap1b had a positive correlation with the TMB and MSI of COAD, and the absolute value of COAD was relatively higher.

## Correlations of Rap1b expression with MMRs and DNA methylation

**Figure 7.**
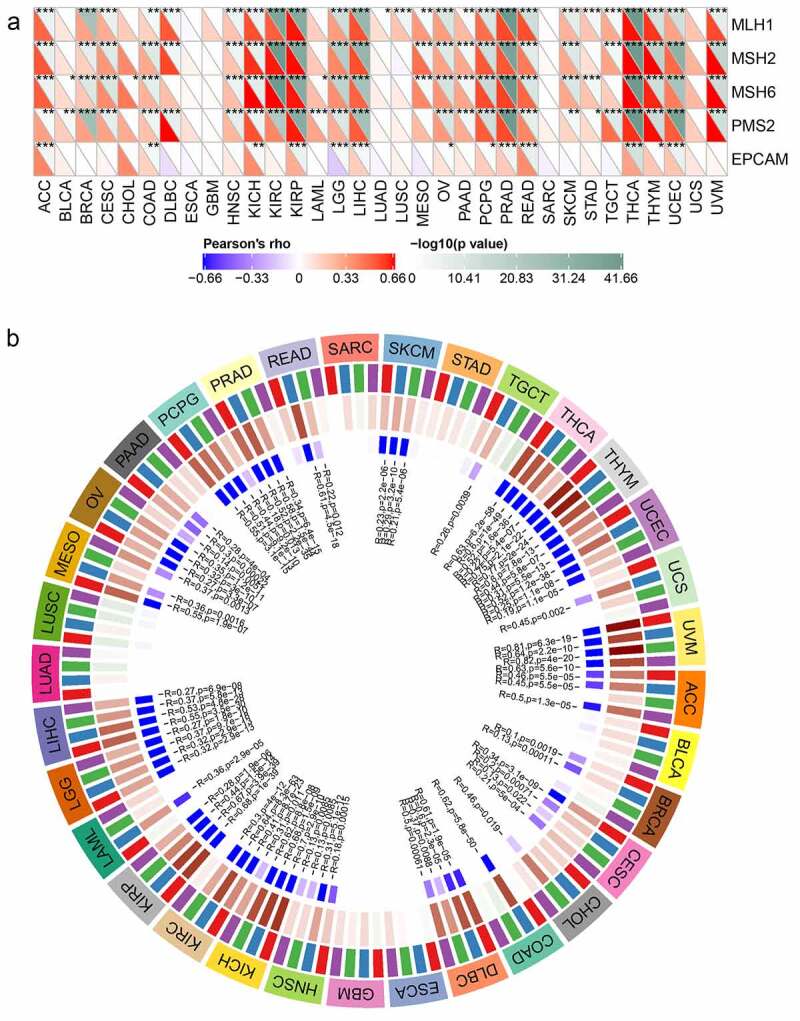
**Relationships between Rap1b, MMRs deficiency and DNA methylation level in different cancers**. (a) The relationship between the *Rap1b* expression of five important MMR related genes (MLH1, MSH2, MSH6, PMS2, EPCAM). The lower triangle of each tile represents the coefficient calculated by Pearson correlation test, and the upper triangle represents the *P*-value after log10 transformation. *P < 0. 05, **P < 0. 01, ***P < 0.001. (b) The relationship between *Rap1b* and four methyltransferases (DNMT1: red, DNMT2: blue, DNMT3A: green, DNMT3b: purple)

MMRs and DNA methylation were widely considered to be important influencing factors of tumor genesis. By studying the correlations between *Rap1b*, MMRs and DNA methylation of specific tumor suppressor genes, the potential mechanism of Rap1b in tumorigenesis could be explored more specifically. Therefore, we analyzed the relationship between Rap1b expression and multiple mature MMR genes (MLH1, MSH2, MSH6, PMS2, and EPCAM). The results showed that in addition to ESCA, GBM, SARC and UCS, Rap1b was significantly associated with MMR gene expression in 29 tumor types. MLH1, MSH2, MSH6, and PMS2 were positively correlated with Rap1b in most of these tumors. Similarly, *Rap1b* expressions in ACC, CDAD, KICH, KIRP, LIHC, OV, PCPG, PRAD, READ, THCA, THYM, and UCEC were positively associated with the five MMR genes, suggesting that Rap1b may play a role in tumors by regulating MMRs (Figure 7a). The expression of 4 methylation transferases (DNMT1, DNMT2, DNMT3A, and DNMT3b) was significantly correlated with Rap1b expression in certain tumor types (UVM, THYM, KICH, KIRP, KIRC, THCA, COAD, DLBC, READ, PRAD, LIHC, UCEC, PCPG, MESO, ACC, CHOL, UCS, LGG, LAML, OV, CESC, PAAD, SKCM, TGCT, HNSC, and BRCA). It is worth noting that the co-expression coefficients of UVM, THYM, KICH, KIRP, KIRC, THCA, COAD, DLBC, and READ were significantly higher, while the co-expression coefficients of other tumors were lower (Figure 7b).

## Discussion

Ras-associated protein1 (RAP1), a member of the Ras small G protein family, is involved in the regulation of signaling pathways including proliferation, differentiation, polarity and apoptosis [26]. *Rap1b* as a new type of extracellular vesicles and granules (EVP) had the potential to become ideal diagnostic biomarker [27]. In this study, we found that the expression level of *Rap1b* varies in different tumors which strongly correlated with prognosis in patients with tumors, and higher expression of *Rap1b* usually was linked to poor prognosis in different datasets. Meanwhile, *Rap1b* was correlated closely with tumor immunity and interacted with various immune cells in different types of cancers. There were significant positive correlations between *Rap1b* expression and ICP, TMB, MSI, MMRs, and DNA methylation. Together, these findings strongly suggest that *Rap1b* expression represent an independent prognostic biomarker for various tumors. Downregulation of *Rap1b* could inhibited the effect of LINC00514 on the proliferation, migration, and invasion in pancreatic cancer cells [28]. Multiple studies have shown that the tumor growth of pancreatic cancer depends on angiogenesis, which affected proliferation and metastasis of solid tumors [29]. These correlations may be the potential reasons for the positive correlation between the high expression of *Rap1b* and the poor prognosis in tumor patients, which suggested that Rap1b could be serve as a prognostic biomarker in various tumors.

The interaction between immune cells and tumor cells in tumor microenvironment (TME) regulated tumor growth, progression and metastasis [16]. Conventionally, the infiltration of immune cells in the TME was a component of an antitumor strategy to avoid tumor cells being killed [30]. *Rap1b* had a certain regulatory effect on immune cells and the deficiency of Rap1b resulted in the fetal lymphatic development defects[20]. In addition, *Rap1b* was essential for splenic B cell proliferation, T cell-dependent humoral immunity, and regulated B cell adhesion and chemotaxis [18]. As the main isotype in NK cells, *Rap1b* could regulate the homing and transportation of NK cells [31]. In this study, we investigated the correlation between *Rap1b* expression and immune cells. We found that *Rap1b* expression had significant correlation with infiltrating levels of B cells in 21 types of cancer, CD4 + T cells in 18 types of cancer, CD8 + T cells in 24 types of cancer, neutrophil in 27 types of cancer, macrophage in 26 types of cancer and dendritic in 26 types of cancer. These results revealed that Rap1b played an important part in recruitment and regulation of immune infiltrating cells in various tumors.

There were plenty of treatments for immune cells in tumors, such as adoptive T cell therapy of transgenic T cells [32], CART cell therapy [33]. In addition, the application of immune checkpoint inhibitors enhanced cancer immunity via blocking immune checkpoint receptors or the ligands, inhibiting tumor metastasis and recurrence, and reducing off-target adverse reactions [34]. Combination therapy with atezolizumab, an anti-programmed cell death ligand 1 (PD-L1) immune checkpoint inhibitor, significantly improve OS in patients with metastatic triple-negative breast cancer [35]. In the case of chemotherapy resistance, the immune checkpoint inhibitor (ICI) can treat some types of tumors with high microsatellite instability and high tumor mutation burden. Multiple studies have found that the overexpression of Rapb in different types of tumors increased the therapeutic resistance to certain anticancer drugs, suggesting that the abnormal expression of *Rap1b* may be related to chemotherapy drug resistance [36,37]. Our study found that *Rap1b* was positively correlated with multiple immune checkpoint genes in various tumors. Therefore, *Rap1b* may be used as a novel anticancer immunotherapy drug target or in combination with known immune checkpoint inhibitors to enhance immune and responses in cancers. In addition, there results indicated that Rap1b might be a promising target in antitumor immunotherapy. Combing chemotherapeutic drugs with *Rap1b* depletion could be used as a new antitumor strategy.

Mismatch repair mainly identified and corrected the errors during DNA replication to ensure the stability of the genome [38,39]. Mismatch repair system is a highly conserved repair mechanism which exists widely in organisms. The mismatch repair proteins cooperate with each other to recognize, remove and repair the mismatched bases. When the mismatch repair system is functionally abnormal, microsatellite sequences are prone to errors in the replication process, leading to mismatches, insertions, and deletions of one or more bases, leading to genomic instability and high mutant phenotypes, and increasing the risk of tumorigenesis [40,41]. In addition, MMR has an effect on repairing DNA replication errors in both normal and cancer cells [42], such as mutations in MMR gens that result in the occurrence of colorectal cancer. In this study, we explored the relationship between five MMRs related genes, including MLH1, MSH2, MSH6, PMS2, EPCAM, and *Rap1b* expression to evaluate tumor somatic mutations. DNA methylation is one of the main forms of epigenetic modification, and DNA methyltransferase (DNMT), the main regulator of DNA methylation, includes *DNMT1, DNMT2, DNMT3a* and *DNMT3b* [43]. Abnormal DNA hypermethylation occured in all stages of tumor genesis and development, and the high expression of DNMT first appeared in the precancerous lesion stage, suggesting that the hypermethylation silencing of various tumor suppressor genes induced by DNMT overexpression and activation was one of the early molecular events in tumor genesis and development [44,45]. Moreover, DNMT was closely related to the clinicopathology and prognosis of tumor patients [46,47]. The results showed that *Rap1b* expression positively correlated with MMRs and DNA methylation, especially in COAD and BRCA, and a higher co-expression coefficient between *Rap1b* and four DNA methyltransferases in various tumors. Therefore, it is reasonable to surmise that *Rap1b* plays an important role in mismatch repair and DNA methylation in pan-cancer and further studies focus on *Rap1b* expression and tumor immunity could help provide new methods of immunotherapy.

However, our study still has some limitations. Firstly, the results suggested that the expression level of *Rap1b* was related to prognosis and immune infiltration in pan-cancer, we still had no direct evidences to confirm Rap1b affected the prognosis of tumors via acting on immune checkpoints or tumor mutations. Second, we evaluated and analyzed via integrating multiple information bases, but a large proportion of the information in the database came from gene chip and sequencing data, and it lacks enough experimental researches and clinical data. It will affected the results to a certain extent, so subsequent experiments *in vivo* or *in vitro* are needed to verify and clarify the correlation of *Rap1b* expression with tumor immune infiltration. Finally, the evaluation of *Rap1b* expression was based on the mRNA level in the above multiple databases, might not reflect the level of functional protein.

## Conclusion

In summary, *Rap1b* expression was correlated with prognosis in 10 types of cancer, especially in KICH, LIHC, PAAD, MESO, and with increased immune infiltration levels of B cells, CD4 + T cells, CD8 + T cells, neutrophils, dendritic cells in various cancers. In addition, there were significant positive correlations between *Rap1b* expression and ICP, TMB, MSI, MMRs, and DNA methylation. Therefore, Rap1b may have an independent role in immune cell infiltration and could represent a unique prognostic biomarker in patients with tumors.

## Data Availability

We confirm that our article contains a Data Availability Statement. All the data in this study are available from TCGA database (https://portal.gdc.cancer.gov), TIMER database (https://cistrome.shinyapps.io/timer/), GTEx database (http://www.gtexportal/), CCLE database (https://portals.broadinstitute.org/ccle).

## Highlights

(1) The expression level of Rap1b varies in different tumors.

(2) The expression level of Rap1b strongly correlated with prognosis in patients with tumors, higher expression of Rap1b usually was linked to poor prognosis in different datasets.

(3) Rap1b was correlated closely with tumor immunity and interacted with various immune cells in different types of cancers.

(4) There were significant positive correlations between Rap1b expression and ICP, TMB, MSI, MMRs and DNA methylation
